# Structural and FTIR spectroscopic studies of matrix-isolated 3-thio-1,2,4-triazole complexes with carbon dioxide. The UV-induced formation of thiol⋯CO_2_ complexes†

**DOI:** 10.1039/d5ra02230d

**Published:** 2025-05-23

**Authors:** Karolina Mucha, Magdalena Pagacz-Kostrzewa, Maria Wierzejewska

**Affiliations:** a Faculty of Chemistry, University of Wrocław F. Joliot-Curie 14 50-383 Wrocław Poland karolina.mucha@uwr.edu.pl

## Abstract

Matrix isolation FTIR spectroscopy was combined with quantum chemical calculations to characterize complexes of 3-thio-1,2,4-triazole (ST) with carbon dioxide. Geometries of the possible 1 : 1 and 1 : 2 complexes were optimized at the DFT (B3LYPD3) level of theory with the 6-311++G(3df,3pd) basis set. The computational results show that ST interacts specifically with carbon dioxide through different hydrogen bond and van der Waals interactions. For the 1 : 1 complexes of the most abundant ST thione tautomer, four stable minima, ST*n*⋯CO_2_, have been located on the potential energy surface. In contrast, for the ST thiol tautomer, three STl⋯CO_2_ structures were optimized. Experimentally, the two most stable 1 : 1 complexes of ST*n* with CO_2_, characterized by the presence of the N–H⋯O hydrogen bridge and an additional S6⋯C10 interaction, were identified in solid argon upon deposition. Annealing of the matrix at 32 K proved that one 1 : 2 structure is also present, resulting from the addition of a second CO_2_ molecule to the 1 : 1 complexes. The laser irradiation at *λ* = 270 nm, apart from generating the thiol tautomer of ST, also leads to the formation of three thiol⋯CO_2_ complexes. Furthermore, the presence of CO_2_ in the argon matrix was found to influence the efficiency of the UV-induced thione–thiol tautomerization, though to a lesser extent than nitrogen. This suggests that while CO_2_ forms stronger intermolecular interactions with ST, its impact on tautomerization kinetics is less pronounced, highlighting the nuanced role of specific gas-phase interactions in modulating photochemical transformations in low-temperature matrices. The findings presented in this work not only enhance the fundamental understanding of weak intermolecular interactions but also provide new insights into the role of CO_2_-specific effects in photochemical and structural transformations of heterocycles.

## Abstract 2

The findings highlight how gas-specific effects modulate molecular reactivity in low-temperature matrices, providing insights into CO_2_ capture and environmental chemistry.

## Graphical abstract

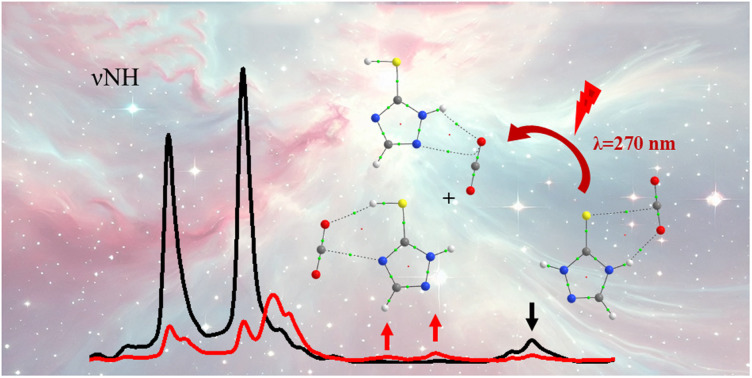

## 1. Introduction

Intermolecular interactions have been the subject of study for many years and have been examined by a wide variety of techniques. The interest in this area is related to the prevalence of this behavior in nature (*e.g.* biochemical processes,^1,2^ the atmosphere,^3,4^ surface science,^5^*etc.*). Moreover, in recent years, there has been a great deal of interest in the interactions of various heterocyclic molecules with carbon dioxide. This is related to the role that CO_2_ plays in the greenhouse effect and the efforts to establish efficient methods for capturing various atmospheric pollutants. Heterocycles, with their diverse electronic and structural properties, offer a promising foundation for developing functional frameworks that selectively bind and sequester CO_2_. A group of compounds that are particularly suited for this purpose are azoles, whose applications have been proven by numerous scientific articles regarding the use of those molecules as MOF, MOP, ZIF, or other frameworks' components.^6–10^ The subject of this work, 3-thio-1,2,4-triazole (ST), makes a relevant candidate for capturing CO_2_ and other pollutants due to the presence of electronegative nitrogen atoms in the ring as well as a thiol group, which creates several possibilities for the acceptor molecule to attach. Another interesting feature of this molecule is its ability to undergo thione–thiol tautomerization, which has been the subject of research due to the importance of heterocyclic tautomerism in various processes related to bioactivity.^11–14^ Recent studies have demonstrated that photo-induced tautomerization, such as keto–enol transitions, can alter the electron density distribution of a molecule and subsequently modify the binding affinity and geometry of CO_2_ complexes, as shown for carbonyl systems under UV exposure.^15^

Based on our previous work on 3-thio-1,2,4-triazole (ST) complexes with dinitrogen,^16^ where we demonstrated different ways of binding N_2_ molecules by the azole and identified particular geometries spectroscopically, we decided to study ST–CO_2_ complexes in a similar way and compare their interaction energies, geometries, and vibrational spectra. Unlike N_2_, which is chemically inert and interacts primarily through dispersion forces and weak hydrogen bonding, CO_2_ exhibits a higher degree of electronic polarizability, leading to stronger and more directional interactions with ST. Therefore, CO_2_ may serve as a modulator of reaction pathways by stabilizing particular conformations or influencing the energy barriers for tautomerization and photolysis. Changes in the matrix environment may influence the tautomeric equilibrium of heterocyclic compounds as well as the efficiency of UV-induced transformations, which has been documented in the literature in previous years.^17–19^ The investigation of ST–CO_2_ complexes introduces new aspects that significantly extend our understanding of intermolecular interactions in matrix isolation.

## 2. Experimental

### 2.1. Theoretical studies

All computations in this work were performed using the Gaussian 16 suite of quantum chemical programs.^20^ The structures of the monomers and the 3-thio-1,2,4-triazole complexes with CO_2_ were created in the GaussView 6.0 program^21^ and then optimized at the B3LYP-D3/6-311++G(3df,3pd) level of theory^22–26^ with the Boys–Bernardi full counterpoise method by Dannenberg *et al.*^27,28^ Interaction energies (*E*_int_) were calculated by subtracting the total electronic energies of the monomers from the energy of the complex while preserving the geometry of the complex. Vibrational wavenumbers were determined using the harmonic approximation to verify that the identified structures correspond to minima on the potential energy surfaces and to monitor spectral changes resulting from the complex formation. To account for anharmonicity, the calculated wavenumbers were scaled by factors of 0.951 for the 4000–2800 cm^−1^ range and 0.982 for the 2800–400 cm^−1^ range. Interactions between ST and CO_2_ in complexes were characterized using topological analysis of electron density (AIM),^29,30^ which was conducted at the B3LYP-D3/6–311++G(3df,3pd) level with the AIM Studio program (Version 19.10.12, Professional).^31^

### 2.2. Matrix isolation experiments

The preparation of cryogenic matrices was carried out using methods and equipment as described in our previous paper on ST complexes.^16^ Carbon dioxide used to prepare CO_2_/Ar matrix gas mixtures was synthesized in the laboratory, and the procedure was described in ref. 32 in section 2.2. The CO_2_/Ar concentrations obtained in the matrix were 1/500 and 1/1000. Photochemical processes were induced in ST/CO_2_/Ar matrices by UV radiation (*λ* = 270 nm) of a pulsed optical parametric oscillator Vibrant (Opotek, Inc.) pumped with a pulsed Nd:YAG laser (Quantel). The progress of the deposition and UV-induced reactions were monitored by recording successive IR spectra.

## 3. Results and discussion

**Fig. 1:**
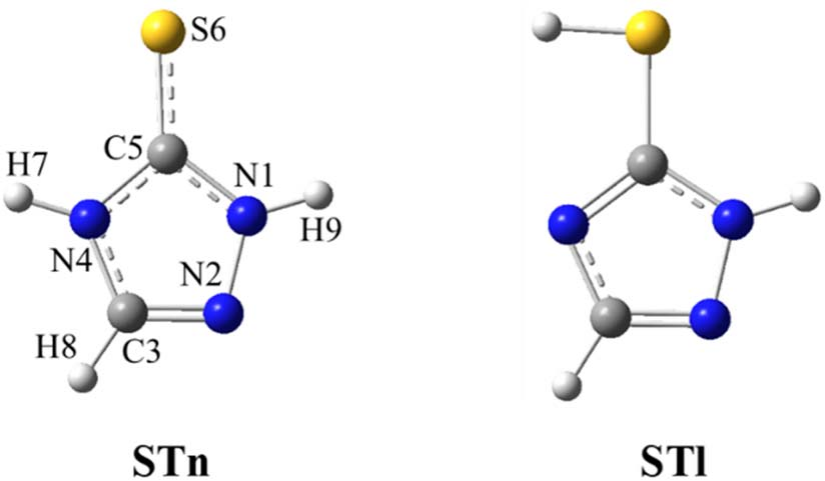
Structures of thione and thiol tautomers of ST.

The monomer of 3-thio-1,2,4-triazole (ST) in the matrix isolation conditions appears in the form of thione tautomer (ST*n*) with trace amounts of the thiol tautomer (STl), as described by Rostkowska *et al.*^33^ Therefore, considering the complexation of ST with carbon dioxide, only those two structures were taken into account. They are presented in Fig. 1 below, together with the adopted numbering of atoms.

### 3.1. Quantum chemical calculations

#### 3.1.1. Complexes of the 1 : 1 stoichiometry

**Fig. 2:**
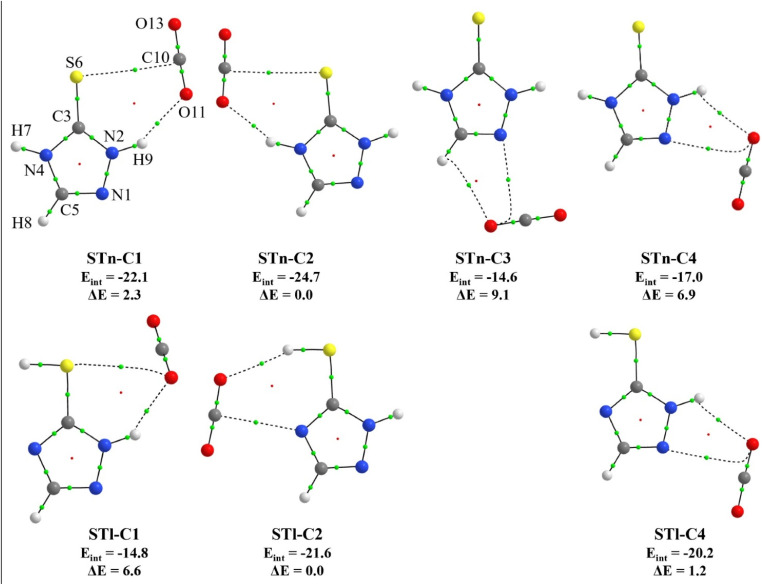
Structures of 1 : 1 complexes of both ST tautomers with CO_2_ molecule. Bond and ring critical points are marked with green and red dots, respectively. The values of interaction energy (*E*_int_) and relative electronic energy (Δ*E*) are given below the structures, both in kJ mol^−1^.

Using the B3LYP-D3/6-311++G(3df,3pd) method, four ST*n*⋯CO_2_ structures and three STl⋯CO_2_ structures were optimized. They are presented in Fig. 2 together with the atom numbering as well as calculated values of the interaction energy *E*_int_ and relative electronic energy Δ*E*. Selected geometric parameters: bond lengths and angle values and key topological data generated by AIM calculations are given in Tables S1 and S2 in the ESI.† Since the thione and thiol forms differ only in the position of one of the hydrogen atoms, the optimized geometries of the complexes of both tautomers are similar. Therefore, it is possible to distinguish analogous structures in both groups. They are marked with the same number (ST*n*–C1 and STl–C1, ST*n*–C2 and STl–C2, ST*n*–C4 and STl–C4). The thiol equivalent of the ST*n*–C3 complex could not be optimized, as it appeared as a very shallow minimum that undergoes a barrierless transformation into the more stable STl–C4 structure. In the first pair of complexes, ST*n*-C1 and STl–C1, the subunits are connected by a hydrogen bond bridge N1–H9⋯O11, the angle of which equals 148.6 and 139.0^0^, respectively. The difference in the values of this angle results from the different position of the CO_2_ molecule relative to the triazole ring. In the structure of ST*n*–C1, it lies in the plane, while in the case of STl–C1 it is tilted out of the plane.

In both complexes, there are also additional van der Waals interactions formed between the sulfur atom of the substituent and one of the atoms of the CO_2_ molecule. In the case of ST*n*–C1, it is a carbon atom, and in STl–C1, it is an oxygen atom. Interestingly, the interaction energy value calculated for the thione form complex is significantly higher than for the second complex (−22.1 kJ mol^−1^ compared to −14.8 kJ mol^−1^). This is most likely because in ST*n*–C1, the geometry is stabilized by interactions involving two atoms of the CO_2_ molecule (C10 and O11), while in the case of STl–C1, it is only an oxygen atom, whose electron density is distributed over two interactions. The next pair of structures are ST*n*–C2 and STl–C2, which are also stabilized by two connections – a hydrogen bond and a van der Waals contact. Due to the presence of two NH groups in ST*n*–C2, the subunits are linked by an N4–H7⋯O11 hydrogen bond and an additional S6⋯C10 interaction. In the case of STl–C2, it is an S6–H7⋯O11 hydrogen bond and an N4⋯C10 interaction. It is worth mentioning that these complexes turned out to be the most stable in their groups (thione and thiol tautomers). The structures ST*n*–C4 and STl–C4 are characterized by the presence of a hydrogen bridge N1–H9⋯O11 and an additional N2⋯O11 connection. Both geometries are planar, and the NHO angle is 120.0 and 120.9^0^, respectively. Despite their very similar structure, the interaction energy values of these complexes differ by 4.2 kJ mol^−1^ in favor of the STl–C4 form. The last of the optimized structures, ST*n*–C3, is the least stable, with the interaction energy equal to −14.6 kJ mol^−1^. It is caused by the fact that, instead of the NH group, the CH group of the ring is involved in the interaction, forming a very weak hydrogen bond.

Worth mentioning is a certain relationship between the length of hydrogen bridges of individual structures and their energetic stability. In the thione tautomer group, the most stable ST*n*–C2 complex has a hydrogen bridge involving the N4–H group. The H7⋯O11 distance in this bridge is smaller than the analogous value of H9⋯O11 in the ST*n*-C1 structure where the N2–H group is involved (2.093 Å compared to 2.149 Å, respectively). This suggests that the hydrogen bonds formed by the N4–H group are stronger than those formed by the N2–H group. This observation is consistent with the results of the structural analysis of ST complexes with N_2_.^16^

To better understand the origin of complex stability, we analyzed the hydrogen bond lengths and AIM topological parameters across all structures. The most stable complexes, ST*n*–C2 and STl–C2, exhibit notably shorter N–H⋯O hydrogen bond distances and higher electron densities at the corresponding bond critical points, indicating stronger and more directional interactions. Furthermore, cooperative van der Waals contacts involving the sulfur atom (*e.g.*, S⋯C interactions) enhance stabilization by supporting the binding geometry. These features correlate well with the calculated interaction energies, highlighting the synergistic role of hydrogen bonding and dispersion forces in determining the relative stabilities of the complexes.

#### 3.1.2. Complexes of the 1 : 2 stoichiometry

**Fig. 3:**
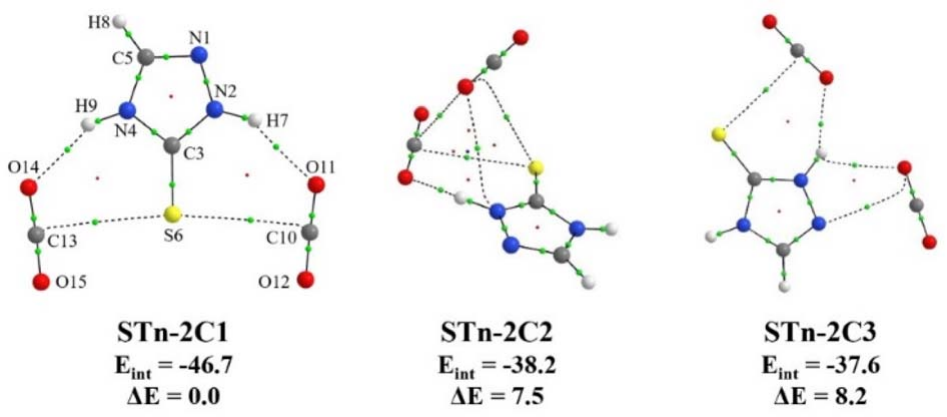
Structures of 1 : 2 complexes of the ST*n* tautomer with CO_2_. Bond, ring, and cage critical points are marked with green, red, and blue dots, respectively. The values of interaction energy (*E*_int_) and relative electronic energy (Δ*E*) are given below the structures, both in kJ mol^−1^.

Although the main goal of the study was to detect and analyze ST–CO_2_ complexes of 1 : 1 composition, the formation of 1 : 2 aggregates could not be excluded under the experimental conditions used. The performed B3LYP-D3/6-311++G(3df,3pd) calculations revealed three stable structures formed between the ST thione tautomer and two CO_2_ molecules. They are presented in Fig. 3, together with the values of interaction energy and relative energy.

Out of three 1 : 2 geometries, the most stable ST*n*–2C1 is characterized by two hydrogen bonds: N2–H9⋯O11 and N4–H7⋯O14, as well as two additional stabilizing van der Waals interactions connecting the S6 atom with the C10 and C13 atoms of two CO_2_ molecules. The H7⋯O14 distance in the hydrogen bridge formed by the N4–H group is smaller than the H9⋯O11 distance of the N2–H9⋯O11 bond (2.155 Å compared to 2.096 Å). This is consistent with the data obtained for ST complexes with CO_2_ of the 1 : 1 stoichiometry. In turn, the ST*n*–2C2 and ST*n*–2C3 complexes are characterized by higher energy values (by 7.5 and 8.2 kJ mol^−1^, respectively), as well as significantly lower interaction energies. The first one has an interesting geometry, in which, according to the AIM results, the arrangement of bond paths allowed for the formation of as many as four rings, which together form a cage. The AIM topological parameters of individual BCPs of the 1 : 2 structures, together with their selected geometric parameters, are available in Table S3 of the ESI.†

### 3.2. Matrix isolation infrared spectra of ST/CO_2_/Ar samples

In agreement with the previously published results^16^ the ST monomers isolated in an argon matrix are present in the ST*n* thione form (see Fig. 1). The introduction of the CO_2_ dopant into the matrix gas resulted in the appearance of a series of bands that had not been present in the spectrum of ST in pure argon. To identify the particular geometries of the 1 : 1 complexes formed between ST and CO_2_ in the matrix, positions of the new bands were compared with the theoretical wavenumbers calculated at the B3LYP-D3/6-311++G(3df,3pd) level. The values of the predicted band shifts of the four optimized 1 : 1 geometries (relative to the analogous monomer absorptions), together with their intensities and the corresponding experimental shifts, are summarized in Table 1.

**Table 1:** Selected shifts of wavenumbers Δ*ν* (cm^−1^) and band intensities (km mol^−1^, in brackets) calculated at the B3LYP-D3/6-311++G(3df,3pd) level for the 1 : 1 complexes of the thione tautomer (ST*n*) with a CO_2_ molecule, compared with the experimental shifts

Theoretical shifts (Δ*ν*)				Mode^16^^,^^a^	Experimental shifts^b^	Assignment
ST*n*–C1	ST*n*–C2	ST*n*–C3	ST*n*–C4			
−10 (84)	−1 (104)	−1 (89)	−10 (67)	νN_2_H	n.o.	
−29 (228)	−53 (249)	0 (103)	−7 (177)	νN_4_H	−26.5	ST*n*–C1
					−52.0	ST*n*–C2
+1 (81)	+2 (61)	0 (50)	+2 (58)	νCN + δNH	+1.0	ST*n*–C1
					+3.0	ST*n*–C2
+4 (402)	+6 (430)	+1 (457)	+1 (507)	νCN + δNH + δ_ring_	+4.5, +6.0	ST*n*–C1
					+7.0	ST*n*–C2
+3 (15)	+5 (16)	−1 (21)	+1 (16)	νCN + δ_ring_	+7.0	ST*n*–C2
					+9.0	ST*n*–C1
−1 (77)	+3 (84)	+1 (100)	+1 (96)	νC = S + νCN + δ_ring_	−1.5	ST*n*–C1
					+2.0	ST*n*–C2
+4 (26)	+5 (34)	+4 (37)	+3 (42)	δ_ring_	+2.5	ST*n*–C1
					+4.5	ST*n*–C2
−33 (89)	−36 (64)	−15 (80)	−13 (71)	δCO_2_	−17.0	ST*n*–C1
					−19.0	ST*n*–C2
+40 (81)	+65 (101)	+3 (138)	+16 (120)	γNH + γ_ring_	+28.5	ST*n*–C1
					+30.5	ST*n*–C2

a Abbreviations: ν – bond stretching, δ – in-plane bending, γ – out-of-plane bending.

b Positions of the ST monomer bands: 3498.0, 3491.0, 1558.0, 1474.5, 1309.0, 1192.5, 932.5, 662.0, 566.0 cm^−1^.

**Fig. 4:**
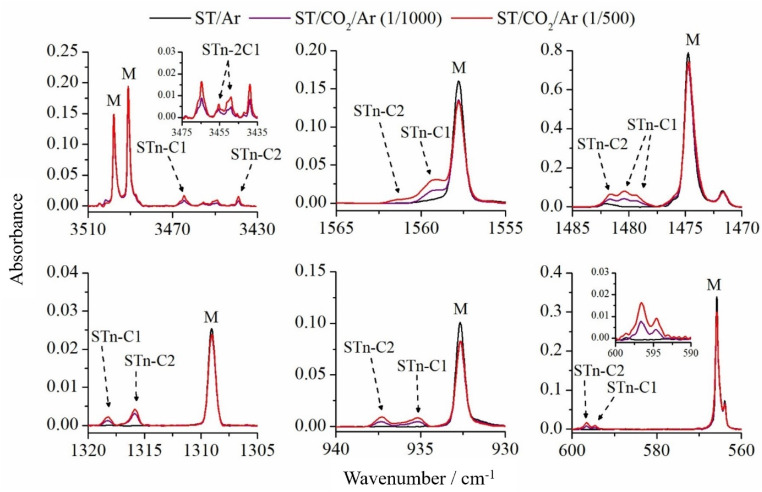
Selected spectral ranges of ST/CO_2_/Ar matrices, where the CO_2_/Ar ratio was 1/500 (red line) and 1/1000 (purple line) and, for comparison, the spectrum of the ST/Ar matrix (black line). The letter M shows the ST monomer bands.

Six selected fragments of the spectra of ST/CO_2_/Ar matrices are presented in Fig. 4 compared with the ST spectrum in pure argon. In the NH stretching mode range (3510–3430 cm^−1^), in addition to two intense absorptions caused by the ν(N_2_H) and ν(N_4_H) vibrations of the ST thione located at 3498.0 and 3491.0 cm^−1^, four new bands at lower wavenumbers appeared as a result of the complexation of the ST monomer with the CO_2_ molecule. Two of them, at 3464.5 and 3439.0 cm^−1^, are characterized by a constant intensity ratio regardless of the CO_2_ concentration in the system, which suggests that they can be assigned to 1 : 1 complexes. The observed wavenumber shifts relative to the ST*n* monomer are equal to −26.5 and −52.0 cm^−1^. On the other hand, the doublet located at 3455.5/3449.0 cm^−1^ is more intense at the CO_2_/Ar mixture concentration of 1/500. This most likely indicates the presence of aggregates of 1 : 2 stoichiometry.

Based on the results of DFT calculations, the two most stable 1 : 1 complexes of ST*n* with CO_2_, namely ST*n*–C1 and ST*n*–C2, are characterized by −29 and −53 cm^−1^ shifts in the ν(N_4_H) stretching region, respectively. These values are in very good agreement with the experimental observation. Both geometries are characterized by the presence of the N–H⋯O hydrogen bridge and an additional S6⋯C10 interaction (see Fig. 2). In the remaining two ST*n*⋯CO_2_ complexes (ST*n*–C3 and ST*n*–C4), their NH groups do not participate in the formation of interactions with carbon dioxide, therefore their theoretical shifts of the νN_4_H mode are much smaller (0 and −7 cm^−1^, respectively).

Other vibrations of the ST molecule are also sensitive to complexation, as shown in the remaining spectral regions of Fig. 4 and Table 1. In each region, new bands are present, shifted by a few cm^−1^ towards higher wavenumbers, and their positions correspond to the predicted shifts for ST*n*–C1 and ST*n*–C2 species. In the range of 1485–1470 cm^−1^, containing the most intense band of the ST monomer originating from complex stretching and deformation vibrations of the triazole ring, the band of the ST*n*–C1 complex appears as a doublet separated by +4.5/+6.0 cm^−1^, and the third band (+7.0) can be assigned to the ST*n*–C2 structure. In the remaining parts of the spectrum, the bands of both complexes appear as singlets. The observed shifts of the modes presented in Table 1 fit very well to the corresponding theoretical values obtained for ST–C1 and ST–C2 structures, indicating that both complexes are present in solid argon. It should be noted that the presence of the ST*n*–C3 complex in the matrix cannot be completely ruled out because the DFT-predicted wavenumber shifts are, in many cases, very small or zero, and the bands originating from this structure can be obscured by the ST monomer peaks. On the other hand, much lower energetic stability of this form (9.2 kJ mol^−1^ difference relative to the most stable complex) indicates that the formation of this structure is unlikely.

**Fig. 5:**
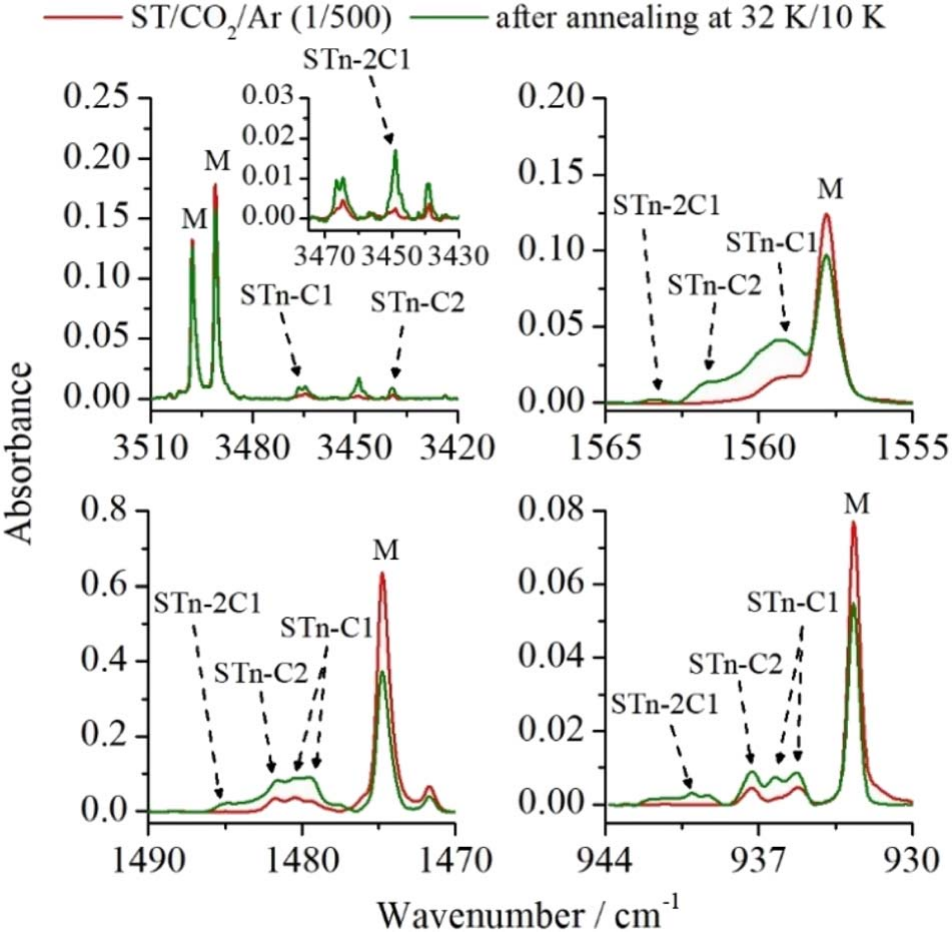
Selected spectral ranges of ST/CO_2_/Ar matrices (CO_2_/Ar = 1/500) after deposition (red line) and after annealing the matrix to 32 K/10 K (green line). The letter M shows the ST monomer bands.

As mentioned earlier, two bands were also formed in the νNH range of the spectrum after deposition, which could be assigned to one of the complexes with stoichiometry higher than 1 : 1. To confirm this interpretation and to reveal more bands originating from these aggregates, the matrix temperature was increased to 32 K and cooled back to 10 K. A change in the intensity of the bands present in the spectrum and the appearance of new ones were observed. As shown in paragraph 3.1.2, the performed calculations reveal three ST*n*⋯(CO_2_)_2_ structures resulting from the addition of a second CO_2_ molecule to the 1 : 1 complexes (see Fig. 2). Fig. 5 shows four spectrum fragments, based on which the analysis and discussion on the structure of 1 : 2 complexes was carried out. The previously unassigned bands in the νNH range are located at 3445.5 and 3449.0 cm^−1^. Upon annealing, the latter was observed to increase its intensity more significantly, which allowed us to assume that both bands originate from the same structure isolated in two different matrix cavities. The second possibility is the presence of overlapping νN_(2)_H and νN_(4)_H bands of one of the 1 : 2 complexes. Comparison of the experimental and theoretical frequencies and intensities allowed us to conclude that the band located at 3449.0 cm^−1^ comes from the νN_(4)_H vibration, and the other from the νN_(2)_H vibration of the ST*n*–2C1 form. This is also confirmed by the fact that in other spectral ranges, no new absorptions were observed that could be assigned to another structure with the 1 : 2 composition. The data is available in Table S4 in the ESI.†

The described analysis at this point does not include complexes of the ST thiol tautomer. This is because this form is present in the matrix in a very small amount after deposition, and the observation of its complexes with the CO_2_ molecule is impossible. However, information on the optimized STl⋯CO_2_ structures, their geometry, energetics, and spectroscopic parameters (see paragraph 3.1.2.) are important in the context of the next paragraph, where the photoinduced tautomerization of ST*n* and its complexes are discussed.

### 3.3. Photochemical generation of the thiol form STl complexes with CO_2_

**Fig. 6:**
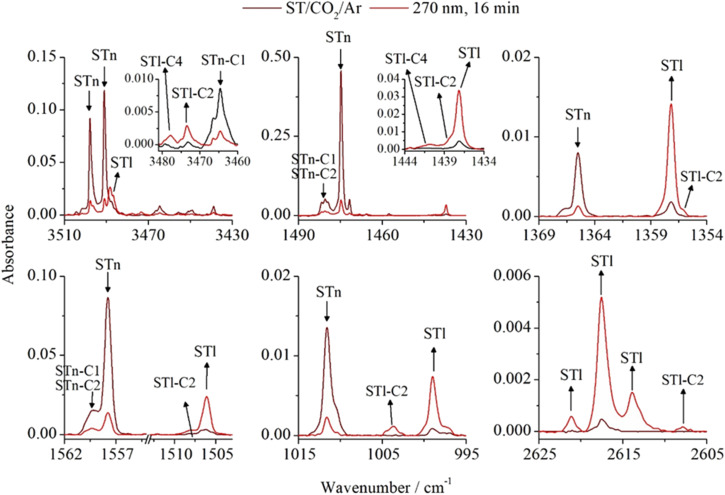
Selected spectral ranges of the ST/CO_2_/Ar matrix (CO_2_/Ar = 1/500) after deposition (burgundy line) and after 16 min of irradiation with radiation of wavelength *λ* = 270 nm (red line).

Laser irradiation of matrices with UV light induced interesting changes in the ST/CO_2_/Ar spectra. Fig. 6 presents several regions of the spectra obtained after 16 min irradiation at 270 nm compared to the spectrum of the matrix after deposition. The formation of the monomer thiol form STl as a result of tautomerization was accompanied by the appearance of additional bands in several spectral ranges. These bands were assigned to the complexes of the thiol form (STl⋯CO_2_), whose structures are presented in Fig. 2. Analyzing individual geometries, it could be predicted that the ST*n*–C1 complex present in the matrix would transform into the STl–C1 form upon irradiation, as this process involves a change in the position of the hydrogen atom H7. In turn, irradiation of the ST*n*–C2 complex will probably lead to the formation of the STl–C2, which is characterized by the lowest energy among all thiol STl⋯CO_2_ complexes. However, despite the lack of the ST*n*–C4 structure after deposition, the appearance of the STl–C4 structure under the influence of irradiation was also considered as a result of attaching a CO_2_ molecule to the STl monomer formed in the matrix cavity.

The above considerations were assessed by comparing theoretical and experimental wavenumber shifts of the new bands (see Table 2), which are located near the analogous absorptions of the STl monomer. In the range of 3510–3430 cm^−1^, bands originating from the following structures are present before irradiation: ST*n*–C1 (3464.5 cm^−1^), ST*n*–C2 (3439.0 cm^−1^), and ST*n*–2C1 (doublet at 3455.5/3449.0 cm^−1^). All of them decreased in intensity upon irradiation and, in addition to a band located at 3488.0 cm^−1^ assigned to the ST1 monomer, a doublet appeared, which is shifted relative to the monomer by −10.0/−15.0 cm^−1^. These values correspond to the theoretical predictions for the νNH vibrations of the STl–C1 and STl–C4 complexes. The NH group of the third structure (STl–C2) is not involved in the interaction, therefore, the calculated νNH shift is equal to zero, and this band is not observed. In the range of 1490–1430 cm^−1^, where there are bands from complex stretching and deformation vibrations of the triazole ring, two new bands of low intensity appear. One of them occurs in the form of a shoulder on the STl monomer band (Δ*ν* = +1.0 cm^−1^), while the other is located away from the monomer absorption by +4.0 cm^−1^. These bands can be assigned to the STl–C2 and STl–C4 structures, respectively, based on the calculated shift values. The remaining spectral regions contain one new band each, whose shifts best correspond to the values predicted for the STl–C2 structure. This suggests that this complex is predominant in the matrix after irradiation. It should also be mentioned that despite the presence of one ST*n* geometry with a stoichiometry of 1 : 2 in the matrix after deposition, no bands were identified in the spectrum after irradiation that could originate from its thiol counterpart, most likely due to the low efficiency of photochemical conversion or a small amount of these species in the matrices.

**Table 2:** Selected shifts of wavenumbers Δ*ν* (cm^−1^) and band intensities (km mol^−1^, in brackets) calculated by the B3LYP-D3/6-311++G(3df,3pd) method for the 1 : 1 complexes of the thiol tautomer ST (STl) with a CO_2_ molecule, compared with the experimental shifts. The values of the wavenumbers of the monomer bands, with respect to which the shifts were calculated, are given in brackets in the rightmost column

Theoretical shifts (Δν̄)			Mode^16^^,^^a^	Experimental shifts^b^	Assignment
STl–C2	STl–C4	STl–C1			
0 (87)	−16 (130)	−7 (147)	νN_2_H	−10.0	STl–C2
				−15.0	STl–C4
−7 (48)	0 (8)	+1 (7)	νSH	−9.5	STl–C2
+2 (43)	−1 (52)	+1 (61)	νCN + δNH + δCN	+2.0	STl–C2
+2 (87)	+4 (131)	+2 (77)	νCN + δNH + δCN + δ_ring_	+4.0	STl–C2
+1 (36)	−3 (39)	−1 (37)	νCN + δ_ring_	−1.0	STl–C2
+4 (40)	+1 (59)	+2 (35)	δCN + δ_ring_	+4.5	STl–C2

a Abbreviations: ν – bond stretching, δ – in-plane bending.

b Positions of the ST monomer bands: 3488.0, 2617.5, 1506.0, 1437.0, 1357.0, 999.0 cm^−1^.

### 3.4. Effect of ST complexation with CO_2_ on the efficiency of thione–thiol tautomerization

**Fig. 7:**
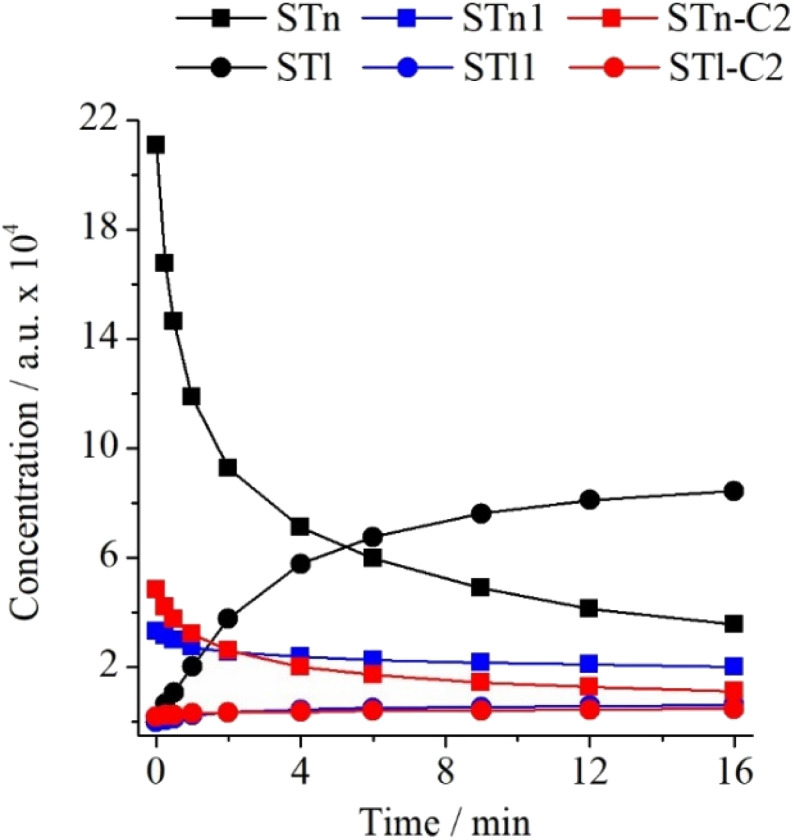
Changes in the intensity of the νN_2_H bands of ST monomers in the argon matrix and their complexes in the matrix doped with N_2_ or CO_2_ as a function of irradiation time at *λ* = 270 nm.

Hydrogen atom transfer upon UV radiation is quite often observed in low-temperature matrices. In their latest review on this topic, Lapinski *et al.*^19^ drew attention to the influence of matrix material on the H-atom transfer phenomenon. What is more, results of our recent paper on ST⋯N_2_ complexes^16^ showed that the efficiency of hydrogen atom transfer in ST complexed with N_2_ is lower than in the monomeric form. Moving forward, it seemed interesting to investigate how the specific 1 : 1 interaction between ST*n* and CO_2_ influences the described thione → thiol transformation. For this purpose, the experimental integrated intensity ratios of the νN_2_H bands were determined for ST*n* and STl monomers as well as for the ST*n*–C2 and STl–C2 complexes. The ST/Ar and ST/CO_2_/Ar spectra recorded before and after 16 min of irradiation with radiation of *λ* = 270 nm were used. As can be seen in Table S5 (ESI),† the νN_2_H band intensity ratio equals 0.400 for monomers and 0.318 for complexes. Thus, the specific interaction of ST with the CO_2_ molecule causes the transfer of the hydrogen atom in the complex to be about 20% less efficient than in the case of the monomer. This difference is smaller than in the case of the interaction with the nitrogen molecule, which indicates that despite the formation of stronger interactions between the ST and CO_2_ subunits, they disturb the kinetics of the photochemical transformation to a lower extent. Changes in the concentration of individual monomers and complexes are presented in Fig. 7.

**Fig. 8:**
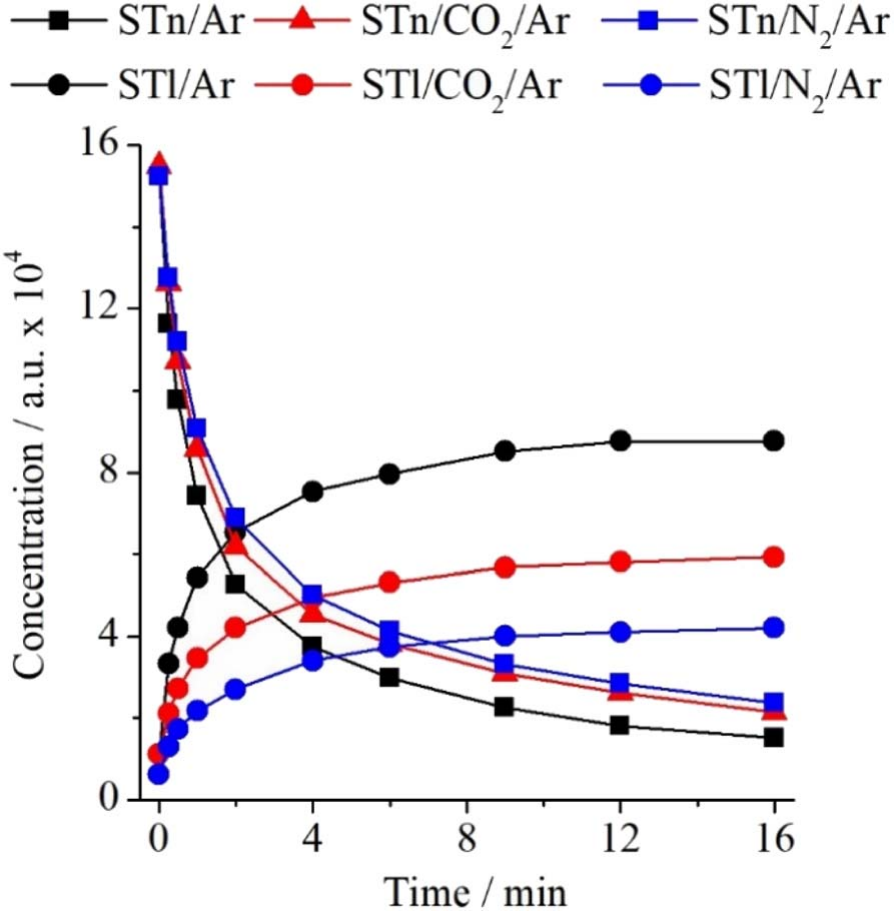
Changes in the intensity of the most intense bands of ST*n* and STl monomers (1474.5 cm^−1^ and 1437.0 cm^−1^, respectively) in ST/Ar, ST/N_2_/Ar, and ST/CO_2_/Ar matrices as a function of irradiation time at *λ* = 270 nm.

Analysis of the ST/Ar and ST/CO_2_/Ar spectra before and after irradiation revealed additional insights into how changes in bulk interactions, caused by the admixture of CO_2_ in the argon matrix, affect the thione → thiol transformation of the ST monomer. The intensity ratios calculated based on the spectra of the ST/Ar and ST/CO_2_/Ar matrices (CO_2_/Ar = 1/500) are presented in Table S6 (ESI).† It presents that, in comparison to the nitrogen admixture, the presence of CO_2_ molecules in the ST*n* environment has a less significant effect on the discussed photochemical reaction in the unbound ST. As shown in Fig. 8 below, one can notice that the decrease of ST*n* and the simultaneous increase of STl are less substantial than in the case of the N_2_-containing matrices.

## 4. Conclusions

Building on our previous work on 3-thio-1,2,4-triazole (ST) complexes with dinitrogen,^16^ the study of ST complexes with carbon dioxide offers a compelling extension to explore the role of non-covalent interactions in atmospheric and environmental chemistry. Unlike N_2_, which primarily interacts *via* weak van der Waals forces and specific hydrogen bonds, CO_2_'s linear geometry and quadrupolar nature allow for diverse interaction modes, including C=O⋯H hydrogen bonding and Lewis acid–base interactions with electron-rich sites on ST. Our findings reveal that the presence of CO_2_ in the matrix influences the photochemical behavior of ST, particularly its thione–thiol tautomerization. While both N_2_ and CO_2_ reduce the efficiency of this transformation compared to the isolated monomer, the effect of CO_2_ is notably less pronounced despite its stronger binding interactions. This suggests that the extent to which a gas-phase species modulates photochemical processes in low-temperature matrices depends not only on interaction energy but also on the geometry and specificity of intermolecular contacts. In this context, the current findings provide important insight into how CO_2_ modulates tautomerization *via* directional, non-covalent interactions. Although ST⋯CO_2_ complexes exhibit stronger binding than their N_2_ counterparts, the reduction in tautomerization efficiency remains modest, underscoring that interaction geometry plays a more decisive role than energetic strength alone. Complexes such as ST*n*–C2, stabilized by hydrogen bonds and S⋯C van der Waals contacts, subtly hinder hydrogen transfer without entirely suppressing reactivity. These results highlight that photochemical reactivity in matrix-isolated systems is governed by the orientation and distribution of interactions, not solely their binding strength. Given the importance of CO_2_ in environmental chemistry and the growing interest in heterocyclic frameworks for CO_2_ capture, the findings presented here not only deepen our understanding of weak intermolecular forces but also shed light on CO_2_-specific effects in photochemical and structural transformations of heterocycles. This study, therefore, expands the scope of previous investigations by demonstrating how differences in gas–matrix interactions can reshape molecular reactivity and photochemistry.

## Data availability

Data for this article, including spectra (.dat) and optimized structures (.log), are available at the Zenodo repository at https://doi.org/10.5281/zenodo.15101158.

## Author contributions

K. Mucha: conceptualization, methodology, data curation, investigation, visualization, writing – original draft, writing – eeview & editing. M. Pagacz-Kostrzewa: investigation, formal analysis, visualization; M. Wierzejewska: writing – original draft, supervision.

## Conflicts of interest

There are no conflicts to declare.

## Supplementary Material

RA-015-D5RA02230D-s001
