# Supplementary material for: Structural and FTIR spectroscopic studies of matrix-isolated 3-thio-1,2,4-triazole complexes with carbon dioxide. The UV-induced formation of thiol⋯CO2 complexes
Source: RSC Adv. 2025 May 23;15(22):17379–87. doi: 10.1039/d5ra02230d (PMC12101105; doi:10.1039/d5ra02230d)
Supplement: RA-015-D5RA02230D-s001 [file RA-015-D5RA02230D-s001.pdf]

Supplementary Information (SI) for RSC Advances.
This journal is © The Royal Society of Chemistry 2025

Supplementary material

# Structural and FTIR spectroscopic studies of matrix-isolated 3-thio-1,2,4-triazole complexes with carbon dioxide. The UV-induced formation of thiol···$CO_2$ complexes

K. Mucha*, M. Pagacz-Kostrzewa, M. Wierzejewska

*Faculty of Chemistry, University of Wrocław, F. Joliot-Curie 14, 50-383 Wrocław, Poland*

* Corresponding author e-mail address: karolina.mucha@uwr.edu.pl

**Table S1.** Interatomic distances (Å), angle values (degrees) and electron density parameters (a.u.) determined for bond critical points (BCP) and ring critical points (RCP) in the complexes of the thione tautomer (STn) with $CO_2$ of the 1:1 stoichiometry calculated at the B3LYP-D3/6-311++G(3df,3pd) level.

| Complex STn···$CO_2$ | Intermolecular parameters[a] | | | AIM parameters | | | |
|---|---|---|---|---|---|---|---|
| | Interatomic distance | | Angle value | BCP | ρ(r) | $\nabla^2$ ρ(r) | Ellipticity |
| | H ··· Y | X ··· Y | X-H ··· Y | | | | |
| **STn-C1** | 2.149 | 3.055 | 148.6 | H9 ··· O11 | 0.014 | 0.053 | 0.025 |
| | | 3.364 | | C10 ··· S6 | 0.007 | 0.025 | 1.965 |
| | | | **RCP** (H9-O11-C10-S6-C5-N1) | 6 at. | 0.005 | 0.019 | |
| **STn-C2** | 2.093 | 3.024 | 152.4 | H7 ··· O11 | 0.015 | 0.060 | 0.024 |
| | | 3.352 | | C10 ··· S6 | 0.008 | 0.025 | 1.786 |
| | | | **RCP** (H7-O11-C10-S6-C5-N4) | 6 at. | 0.005 | 0.020 | |
| **STn-C3** | 2.676 | 3.184 | 108.4 | H8 ··· O11 | 0.006 | 0.025 | 1.003 |
| | | 3.026 | | O11 ··· N1 | 0.009 | 0.036 | 1.419 |
| | | | **RCP** (H8-O11-N2-C3) | 4 at. | 0.006 | 0.027 | |
| **STn-C4** | 2.401 | 3.032 | 120.0 | H9 ··· O11 | 0.009 | 0.037 | 0.239 |
| | | 3.023 | | N2 ··· O11 | 0.010 | 0.036 | 1.278 |
| | | | **RCP** (H9-O11-N2-N1) | 4 at. | 0.008 | 0.035 | |

[a] X = N, C or S atom, Y = N, C or O atom.

**Table S2.** Interatomic distances (Å), angle values (degrees) and electron density parameters (a.u.) determined for bond critical points (BCP) and ring critical points (RCP) in the complexes of the thiol tautomer (STl) with $CO_2$ of the 1:1 stoichiometry, calculated at the B3LYP-D3/6-311++G(3df,3pd) level.

| Complex STl···$CO_2$ | Intermolecular parameters[a] | | | AIM parameters | | | |
|---|---|---|---|---|---|---|---|
| | Interatomic distance | | Angle value | BCP | ρ(r) | $\nabla^2$ ρ(r) | Ellipticity |
| | H ··· Y | X ··· Y | X-H ··· Y | | | | |
| **STl-C1** | 2.298 | 3.128 | 139.0 | H9 ··· O11 | 0.010 | 0.039 | 0.050 |
| | | 3.434 | | O11 ··· S6 | 0.006 | 0.020 | 0.470 |
| | | | **RCP** (H9-O11-S6-C5-N1) | 5 at. | 0.005 | 0.021 | |
| **STl-C2** | 2.374 | 3.644 | 156.3 | H7 ··· O11 | 0.009 | 0.032 | 0.003 |
| | | 2.851 | | C10 ··· N4 | 0.011 | 0.042 | 1.408 |
| | | | **RCP** (H7-O11-C10-N4-C5-S6) | 6 at. | 0.005 | 0.020 | |
| **STl-C4** | 2.363 | 3.007 | 120.9 | H9 ··· O11 | 0.010 | 0.039 | 0.172 |
| | | 3.013 | | O11 ··· N2 | 0.010 | 0.037 | 1.391 |
| | | | **RCP** (H9-O11-N2-N1) | 4 at. | 0.008 | 0.037 | |

[a] X = N, C or S atom, Y = N, C or O atom.

**Table S3.** Interatomic distances (Å), angle values (degrees) and electron density parameters (a.u.) determined for bond critical points (BCP) and ring critical points (RCP) in the complexes of the thione tautomer (STn) with $CO_2$ of the 1:2 stoichiometry, calculated at the B3LYP-D3/6-311++G(3df,3pd) level.

| Complex STn···$(CO_2)_2$ | Intermolecular parameters[a] | | | AIM parameters | | | |
|---|---|---|---|---|---|---|---|
| | Interatomic distance | | Angle value | BCP | ρ(r) | $\nabla^2$ ρ(r) | Elipticity |
| | H ··· Y | X ··· Y | X-H ··· Y | | | | |
| **STn-2C1** | 2.095 | 3.026 | 152.7 | H7 ··· O14 | 0.015 | 0.060 | 0.024 |
| | 2.152 | 3.058 | 148.7 | H9 ··· O11 | 0.013 | 0.052 | 0.024 |
| | | 3.364 | | S6 ··· C13 | 0.008 | 0.025 | 1.841 |
| | | 3.354 | | S6 ··· C10 | 0.007 | 0.025 | 1.992 |
| | **RCP1** (H9-O11-C10-S6-C3-N2) | | | 6 at. | 0.005 | 0.019 | |
| | **RCP2** (H7-O14-C13-S6-C3-N4) | | | 6 at. | 0.005 | 0.020 | |
| **STn-2C2** | 2.170 | 3.069 | 147.6 | H9 ··· O11 | 0.013 | 0.048 | 0.042 |
| | | 2.991 | | C10 ··· O14 | 0.006 | 0.028 | 3.467 |
| | | 3.449 | | S6 ··· C10 | 0.006 | 0.021 | 2.507 |
| | | 3.532 | | S6 ··· O14 | 0.007 | 0.023 | 1.510 |
| | | 3.362 | | N2 ··· O14 | 0.005 | 0.017 | 0.620 |
| | **RCP1** (H9-O11-C10-S6-C3-N2) | | | 6 at. | 0.004 | 0.018 | |
| | **RCP2** (N2-O14-S6-C3) | | | 4 at. | 0.004 | 0.016 | |
| | **RCP3** (O14-N2-H9-O11-C10) | | | 5 at. | 0.003 | 0.014 | |
| | **RCP4** (O14-S6-C10) | | | 3 at. | 0.003 | 0.013 | |
| **STn-2C3** | 2.477 | 3.071 | 117.2 | H9 ··· O11 | 0.008 | 0.032 | 0.366 |
| | 2.180 | 3.070 | 146.2 | H9 ··· O14 | 0.013 | 0.049 | 0.019 |
| | | 3.354 | | S6 ··· C13 | 0.008 | 0.025 | 1.939 |
| | | 3.010 | | N1 ··· O11 | 0.010 | 0.037 | 1.265 |
| | **RCP1** (H9-O14-C13-S6-C3-N2) | | | 6 at. | 0.005 | 0.019 | |
| | **RCP2** (H9-O11-N2-N1) | | | 4 at. | 0.007 | 0.033 | |

[a] X = N or S atom, Y = N, C or O atom.

**Table S4.** Selected shifts of wavenumbers Δν ($cm^{-1}$) and band intensities (km $mol^{-1}$, in brackets) calculated at the B3LYP-D3/6-311++G(3df,3pd) level for 1:2 complexes of the thione tautomer (STl) with a $CO_2$ molecule, compared with the experimental shifts.

| Theoretical shifts (Δν) | | | Mode [16][a] | Experimental shifts[b] | Assignment |
|---|---|---|---|---|---|
| STn-2C1 | STn-2C2 | STn-2C3 | | | |
| -39 (202) | -11 (85) | -11 (89) | νN2H | -41.5 | STn-2C1 |
| -44 (276) | -30 (221) | -44 (235) | νN4H | -42.0 | STn-2C1 |
| +4 (89) | 0 (85) | +3 (84) | υCN + δNH | +5.5 | STn-2C1 |
| +10 (390) | +6 (364) | +5 (484) | υCN + δNH + $\delta_{ring}$ | +10.5 | STn-2C1 |
| +9 (29) | +5 (25) | +7 (36) | $\delta_{ring}$ | +7.5 | STn-2C1 |
| +48 (0) | +12 (20) | +15 (43) | γNH + $\gamma_{ring}$ | n.o. | STn-2C1 |

[a] Abbreviations: ν - bond stretching, δ - in-plane bending, γ - out-of-plane bending.
[b] Positions of the ST monomer bands: 3498.0, 3491.0, 1558.0, 1474.5, 932.5, 566.0 $cm^{-1}$.

**Table S5**. Experimental and theoretical (B3LYP-D3/6-311++G(3df,3pd)) values of the νN2H band intensities for both ST monomers and their respective $N_2$ and $CO_2$ complexes used to estimate the efficiency of photoinduced hydrogen atom transfer.

| Species | $I_{theo}$ | Integrated intensity | | $I_{exp}/I_{theo}$ x$10^4$ | |
|---|---|---|---|---|---|
| | | t = 0 min | t = 16 min | t = 0 min | t = 16 min |
| STn monomer | 101 | 0.213 | 0.036 | 21.09 | 3.56 |
| STl monomer | 90 | 0.0015 | 0.076 | 0.17 | 8.44 |
| **$N_2$ complexes [16]** | | | | | |
| STn2 complex | 255 | 0.084 | 0.051 | 3.29 | 1.86 |
| STl2 complex | 167 | 0.001 | 0.011 | 0.00 | 0.65 |
| **$CO_2$ complexes** | | | | | |
| STn-C2 complex | 249 | 0.010 | 0.0035 | 0.402 | 0.14 |
| STl-C2 complex | 87 | 0.0005 | 0.0011 | 0.057 | 0.128 |

Intensity ratio for the monomer: STl (16 min)/STn (0 min) = 0.400
Intensity ratio for the $N_2$ complex: STl2 (16 min)/STn2 (0 min) = 0.197
Intensity ratio for the $CO_2$ complex: STl-C2 (16 min)/STn-C2 (0 min) = 0.318

**Table S6.** Experimental and theoretical (B3LYPD3/6-311++G(3df,3pd)) intensities of the most intense bands of STn and STl monomers (1474.5 $cm^{-1}$ and 1437.0 $cm^{-1}$, respectively) before and after irradiation, in ST/Ar and ST/$CO_2$/Ar matrices.

| **Species** | $I_{theor}$ | **Integrated intensity** | | $I_{exp}/I_{theor}$ **x10$^4$** | |
|---|---|---|---|---|---|
| | | **t = 0 min** | **t = 16 min** | **t = 0 min** | **t = 16 min** |
| **ST/Ar matrix** | | | | | |
| STn | 420 | 0.64 | 0.063 | 15.24 | 1.50 |
| STl | 81 | 0.009 | 0.071 | 1.11 | 8.76 |
| **ST/$N_2$/Ar matrix [16]** | | | | | |
| STn | 420 | 0.65 | 0.09 | 15.48 | 2.14 |
| STl | 81 | 0.009 | 0.048 | 1.11 | 5.92 |
| **ST/$CO_2$/Ar matrix** | | | | | |
| STn | 420 | 0.64 | 0.10 | 15.25 | 2.36 |
| STl | 81 | 0.005 | 0.034 | 0.62 | 4.21 |

Intensity ratio for monomer in Ar matrix: STl (16 min)/STn (0 min) = 0.548
Intensity ratio for monomer in $N_2$/Ar matrix: STl (16 min)/STn (0 min) = 0.382
Intensity ratio for monomer in $CO_2$/Ar matrix: STl (16 min)/STn (0 min) = 0.452
